# Decolorization of Anthraquinonic Dyes from Textile Effluent Using Horseradish Peroxidase: Optimization and Kinetic Study

**DOI:** 10.1155/2015/371625

**Published:** 2015-01-19

**Authors:** Nataša Ž. Šekuljica, Nevena Ž. Prlainović, Andrea B. Stefanović, Milena G. Žuža, Dragana Z. Čičkarić, Dušan Ž. Mijin, Zorica D. Knežević-Jugović

**Affiliations:** ^1^Faculty of Technology and Metallurgy, University of Belgrade, Karnegijeva 4, 11000 Belgrade, Serbia; ^2^Innovation Center, Faculty of Technology and Metallurgy, University of Belgrade, Karnegijeva 4, 11000 Belgrade, Serbia

## Abstract

Two anthraquinonic dyes, C.I. Acid Blue 225 and C.I. Acid Violet 109, were used as models to explore the feasibility of using the horseradish peroxidase enzyme (HRP) in the practical decolorization of anthraquinonic dyes in wastewater. The influence of process parameters such as enzyme concentration, hydrogen peroxide concentration, temperature, dye concentration, and pH was examined. The pH and temperature activity profiles were similar for decolorization of both dyes. Under the optimal conditions, 94.7% of C.I. Acid Violet 109 from aqueous solution was decolorized (treatment time 15 min, enzyme concentration 0.15 IU/mL, hydrogen peroxide concentration 0.4 mM, dye concentration 30 mg/L, pH 4, and temperature 24°C) and 89.36% of C.I. Acid Blue 225 (32 min, enzyme concentration 0.15 IU/mL, hydrogen peroxide concentration 0.04 mM, dye concentration 30 mg/L, pH 5, and temperature 24°C). The mechanism of both reactions has been proven to follow the two substrate ping-pong mechanism with substrate inhibition, revealing the formation of a nonproductive or dead-end complex between dye and HRP or between H_2_O_2_ and the oxidized form of the enzyme. Both chemical oxygen demand and total organic carbon values showed that there was a reduction in toxicity after the enzymatic treatment. This study verifies the viability of use of horseradish peroxidase for the wastewaters treatment of similar anthraquinonic dyes.

## 1. Introduction

Synthetic dyes are a major class of pollutants in wastewater from various industries such as textile, paper, food, plastics, and cosmetics. Today, over 100,000 different dye structures have been synthesized and more than 0.7 million tons of dyestuff are produced annually [1]. Dyes represent a very large and complex group of organic compounds, which differ in their origin, chemical and/or physical properties, and characteristics related to the application process. They are broadly classified according to their application (the dyeing method) into groups as acid or basic dyes, disperse dyes, direct dyes, mordant dyes, reactive dyes, vat dyes, and so forth, or on the bases of their chemical structure as azo dyes, anthraquinonic dyes, carotenoid dyes, xanthene dyes, triphenylmethane dyes, phthalocyanine, and so forth. Up to 50% of the dyes used in textile dyeing may remain unfixed to the fiber and contaminates the industrial wastewater [2].

In wastewaters, dyes are easily noticeable since they can be observed at lower concentrations starting from 1 mg/L [2]. In addition, the effects caused by other pollutants in textile wastewater and the presence of very small amounts of dyes in the water, which are nevertheless highly visible, seriously affect the aesthetic quality and transparency of lakes, rivers and others, leading to damage to the aquatic environment and formation of dead deoxygenated zones in seas and oceans [3, 4]. The more complex environmental problems associated with the textile wastewaters, besides the coating, are due to wide utilization of carcinogenic or mutagenic dyes, which are resistant to acid and basic conditions, bleaching agents, light degradation, and others [5, 6].

Anthraquinonic dyes are a major class of these environmental colored pollutants due to their recalcitrance and high toxicity and their environmental release by diverse industries [7]. Many of anthraquinonic dyes are mutagenic and even carcinogenic and pose a serious threat to living organisms [8, 9]. For example, amongst two azo- (Reactive Orange 16; Congo Red) and two anthraquinonic dyes (Remazol Brilliant Blue R (RBBR); Disperse Blue 3 (DB3)) tested, the anthraquinone DB3 dye has been proven to be the most toxic in the bacterial, algal, and protozoan tests, exhibiting mutagenic effects after metabolic activation in vitro in all* S. typhimurium* strains used [10]. Also, they are very resistant to degradation due to their fused aromatic structures and remain colored for a long time, which makes them obligatory to remove from industrial effluents before being discharged into the environment. Therefore, two acid anthraquinonic dyes, C.I. Acid Blue 225 and C.I. Acid Violet 109, widely used for wool, polyamide, silk, and the blended fibers dyeing and printing, were selected as models for current study.

Various strategies have been used to remove dyes from textile wastewater and reduce the cost of the overall process including chemical oxidation, physicochemical techniques (coagulation/flocculation, adsorption, and reverse osmosis), electrochemical or microbial decolorization, and, most recently, the use of various enzymes. The use of physical/chemical methods has numerous drawbacks such as: economical unwarrancy (spending a huge amount of chemicals and energy), incomplete dye removal, generating a large amount of mud that can cause a secondary pollution problem, and price augmentation of these methods which is a result of implementation of complicated processes [11]. In contrast to physical/chemical methods, certain biological methods have been developed and have shown themselves as economically more available, considerably more efficient, and ecofriendly for the removal of numerous toxic pollutants [12–14]. Especially, there is growing interest to the enzyme degradation of dyes due to several advantages such as greater specificity, capability to operate over a broad concentration range of contaminants, better standardization, easy handling and storage, and no dependence on bacterial growth rates [15, 16]. Enzymes can act on specific recalcitrant pollutants to remove them by precipitation or transformation to other products. Amongst oxidoreductive enzymes that are involved in dye decolorization, peroxidases such as horseradish peroxidase (HRP), citochrome C peroxidase isolated from yeast, chloroperoxidase isolated from* Caldariomyces fumago*, several fungal lignin peroxidase (LiP) and manganese peroxidase (MnP) such as LiP from* Phanerochaete chrysosporium*, and soybean peroxidase have been reported as excellent oxidant agents to degrade dyes in the presence of hydrogen peroxide [17–21].

Peroxidases (EC 1.11.1.x) are chem consisting proteins (metaloproteins) which use hydrogen peroxide or other organic peroxides as electron acceptors to catalyze the oxidation of a wide range of substrates. Especially, HRP has shown potential for use as a bioremediation catalyst because of its ability to oxidize a large number of dyes even in the presence of contaminants commonly found in wastewaters, flexibility to function at wide ranges of temperature, pH and salinity, ready availability, and relatively low cost [20, 22–24]. Although HRP is highly specific to its peroxide substrate, of which H_2_O_2_ is the most common, it shows broad substrate specificity toward its hydrogen donor substrate, degrading also other aromatic compounds, such as phenol, estrogens, *p*-chlorophenol, and others [25]. Previously, several studies showed that HRP efficiently cleaved aromatic azo compounds in the presence of H_2_O_2_ and degraded and precipitated industrially important azo dyes [23, 26, 27]. However, the studies concerning HRP-catalyzed degradation of anthraquinonic dyes are rather scarce [7].

Dye structure has been shown to have significant effect on the decolorization ability of HRP. Although anthraquinonic dyes account for about 15% colorants, there are only few papers dealing with their degradation by HRP-catalyzed reaction. A better understanding of the mechanism and kinetics of this process would help in the design of a suitable reactor system through process development, optimization, and scale-up.

The aim of this study is to examine the feasibility of using the HRP to remove two selected anthraquinonic dyes from wastewaters. The influence of parameters such as enzyme concentration, dye concentration, hydrogen peroxide concentration, pH of aqueous solution, and temperature was examined in order to determine the optimal conditions for decolorization of anthraquinonic dyes. The present work also focuses on kinetic investigations based on the degradation of the anthraquinonic model dyes by this versatile enzyme.

## 2. Materials and Methods

### 2.1. Materials

Anthraquinonic dyes used in this paper, C.I. Acid Blue 225 (AB 225) and C.I. Acid Violett 109 (AV 109), are obtained from Lanaset (Lanaset Violet B, Lanaset Blue 2R) and their characteristics are presented in Table 1. HRP peroxidase (EC 1.11.1.7; donor: hydrogen peroxide oxidoreductase) with a specific activity of 250 purpurogallin units per mg was purchased from Sigma-Aldrich (St. Louis, MO, USA). All aqueous solutions were prepared in doubly distilled water. The concentration of hydrogen peroxide solutions was determined several times using its molar absorption coefficient (*ε* = 43.6 M^−1^ cm^−1^) at *λ* = 240 nm by dilution of the supplied H_2_O_2_ (30% v/v) solution. Other chemicals used in this work were of commercial analytical grade.

### 2.2. Decolorization Experiments

In order to optimize process parameters for dye decolorization from textile wastewater with HRP, the influence of enzyme concentration, dye concentration, hydrogen peroxide concentration, pH, and temperature was examined for both dyes. The dye (AV 109 or AB 225) was prepared in distilled water or appropriate buffer at concentration of 10–50 mg/L and a selected quantity of hydrogen peroxide was added. The solution was allowed to achieve thermal equilibrium, prior to reaction initiation and was stirred at 100 rpm using magnetic stirring bars. Reactions were initiated by the addition of HRP in order to minimize any potential inactivation of the enzyme due to extreme pH values and peroxide and aliquots of 3 mL volume, were taken from the reaction mixture in defined time intervals and submitted to analytical control. Experiments were made in triplicate, and standard deviations were calculated. The investigated range of the reaction parameters selected in this study is given in Table 2.

The preliminary experiments showed that the HRP alone did not decolorize solutions of both dyes. Also, the solutions of both dyes appeared to be stable upon exposure to H_2_O_2_ alone, revealing that the decolorization was a result of H_2_O_2_-dependent enzymatic reaction.

### 2.3. Initial Kinetic Study

The initial enzyme kinetics was examined in a series of batch reactors by measuring the initial reaction rate of the reaction using different concentrations of dyes and H_2_O_2_. The initial rate was each time determined from the slope of the reaction progress curve at the beginning of the reaction by linear regression. Experiments for each hydrogen peroxide concentration were performed at final concentrations of AV 109 and AB 225 of 0.018 mM and 0.029 mM, respectively. The influence of the AV 109 initial concentration was tested in the range of 0.012 to 0.043 mM at fixed hydrogen peroxide concentration of 0.06 mM, whereas the influence of the AB 225 initial concentration was varied in the range of 0.015 to 0.051 mM at fixed hydrogen peroxide concentration of 0.1 mM. All other parameters were kept constant (for AV 109 solution: pH 5.25, enzyme concentration 0.15 IU/mL, and temperature 24°C; for AB 225 solution: pH 5.5, enzyme concentration 0.04 IU/mL, and temperature 24°C). Each reaction was done in duplicate and standard deviations were less than 5%. The initial kinetic data obtained for HRP-catalyzed oxidation of both dyes were fitted to the two-substrate kinetic models, namely, the ping-pong (three parameters) and the ping-pong kinetic model that included H_2_O_2_ or dye inhibition (four parameters) using Matlab software.

### 2.4. Determination of the Dye Decolorization Percentage

The efficiency of the enzymatic treatment was evaluated by monitoring the decolorization of dyes at their maximum absorption wavelength with a UV-Vis spectrophotometer (UV Shimadzu 1700, Shimadzu Corporation, Kyoto, Japan). For this purpose, a solution of 30 mg/L concentration of dyes was scanned over a wavelength range of 200–800 nm and optimum wavelength was determined (*λ*_max⁡_—628 nm, absorbance—0.265 for AB 225, and *λ*_max⁡_—590 nm, absorbance—0.319 for AV 109). The calculated molar extinction coefficients were 11,019 L mmol^−1^ cm^−1^ and 6,053 L mmol^−1^ cm^−1^ for AV 109 and AB 225, respectively.

The decolorization in percentage for both dyes was determined, according to the equation [28]

(1) $$\begin{array}{c} \text{Decolorization}\left(\%\right)=\left[\frac{\left(A_{0}-A_{t}\right)}{A_{0}}\right]\times 100, \end{array}$$

where *A*_0_ is the initial absorbance of untreated dye solutions (control) and *A*_*t*_ is the absorbance of dye solutions after enzymatic treatment.

Experiments were made in triplicates, and standard deviations were calculated.

### 2.5. Determination of the Chemical Oxygen Demand (COD) and Total Organic Carbon (TOC)

In order to estimate the amount of organic matter in wastewater, the COD was examined. The test measures the amount of oxygen required for chemical oxidation of organic matter in the sample to carbon dioxide and water. COD was examined using the closed reflux method. The samples for COD experiment were prepared under previously determined conditions (for AV 109 dye: pH 4.0, temperature 24°C, hydrogen peroxide concentration 0.4 mM, dye concentration 30 mg/L, and enzyme concentration 0.15 IU/mL; for AB225 dye: pH 5.0, temperature 24°C, hydrogen peroxide concentration 0.04 mM, dye concentration 30 mg/L, and enzyme concentration 0.15 IU/mL). Briefly, 2 mL of the sample is pippeted into vials (10 mL capacity and 19 mm diam.) containing 0.9 mL digestion solution (10.216 g K_2_CrO_7_, 167 mL conc. H_2_SO_4_, and 33.3 g HgSO_4_ in 1 L distilled water) and 2.1 mL sulfuric acid reagent (1 kg conc. H_2_SO_4_ and 5.5 g Ag_2_SO_4_). Water was used as the blank. The mixture is refluxed by COD reactor (2 h, 148–150°C) and then cooled (45 min). The COD determination is made with the spectrophotometer (HANNA, HI 83099).

Content of the total organic carbon was measured using standard SRPS ISO method 8245:2007 with the TOC-VCPA analyzer (Shimadzu Corporation).

## 3. Results and Discussion

### 3.1. Effect of Time on the Dye Decolorization

**Figure 1:**
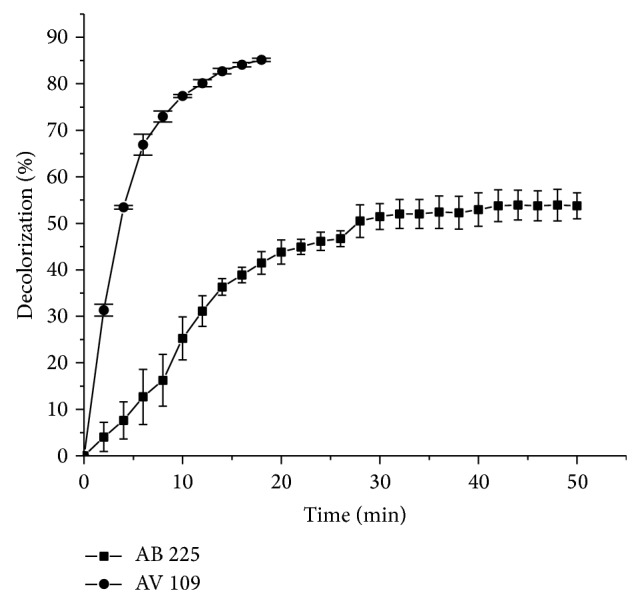
Time course of decolorization of AV 109 (circle) and AB 225 (square) dyes at concentration of 30 mg/L. Other reaction conditions for AV 109 dye: peroxide concentration 2 mM, pH 5.25, enzyme concentration 0.15 IU/mL, temperature 24°C; for AB 225 dye: hydrogen peroxide concentration 1 mM, pH 5.5, enzyme concentration 0.04 IU/mL, and temperature 24°C.

In this study the ability of HRP to decolorize two synthetic anthraquinonic dyes was investigated. The time course of dyes' decolorization by HRP is shown in Figure 1.

The time course of decolorization was apparently different for two dyes. Specifically, following 15 and 32 minutes of incubation, the decolorization percentage of AV 109 and AB 225 was 85.16% and 50.63%, respectively. After mentioned time, there was no significant augmentation in the dye decolorization percentage with increasing contact time. The enzymatic process appeared to lead to a rather high decolorization percentage in the case of AV 109 for a short reaction time, revealing a rather high HRP decolorization efficiency toward this anthraquinonic dye. In the case of other oxidases such as laccase, the decolorization process of an anthraquinonic dye as a model was not observed in the absence of a small molecular weight redox mediator [29].

On the other hand, decolorization of this important class of recalcitrant anthraquinone-type dyes used in the textile industry has been verified by using laccase extracted from* Polyporus* sp. S133, but the reported system required longer reaction times. Specifically, the decolorization percentage was 26 and 60% after 24 and 48 h of incubation, respectively, and with* N*-hydroxybenzotriazole as a redox mediator it increased by 20% [30]. By comparison, other dyes such as di-azo dye Acid Black 10 BX have been completely decolorized at dye concentration of 20 mg/L with 2.205 IU/mL of HRP activity and a 1 h treatment at dye concentration of 20 mg/L after 45 minutes [23].

**Figure 2:**
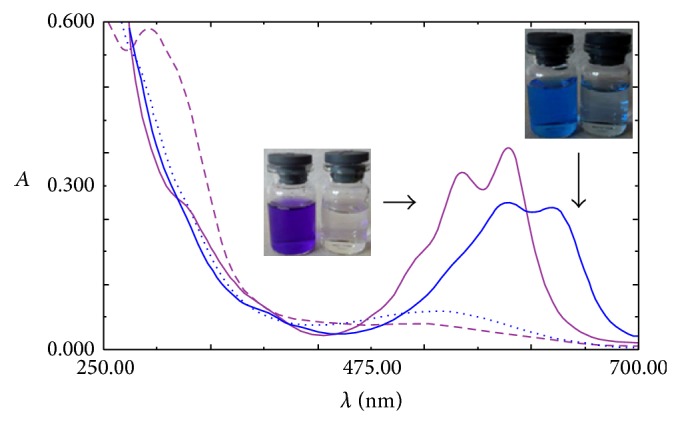
Absorption spectra before (solid lines) and after enzymatic decolorization experiment (dotted lines) for AB 225 (blue) and AV 109 (violet).

It appeared that the affinity of HRP for AV 109 is higher than that for AB 225, suggesting that the dye structure has an important effect on the HRP activity through steric effects, electronic distribution or charge distribution. In addition, the ionisation potential could play an important role [31]. In this work, we selected two anthraquinonic dyes with similar chemical structures. AB 225 is a typical anthraquinonic dye used in the textile industry with a common* para*-diaminoanthraquinone sulfonated moiety substituted on the* para*-amino group. In addition, AB 225 have double bond on amide group. However, AV 109 lacks one amino group while other amino group is substituted with similar moiety. In para position to the substituted amino group, AV 109 have sulphonated phenoxy moiety. To our knowledge, there are no examples of the use of HRP as the catalyst for decolorization of these anthraquinonic dyes. Change in absorption spectrum of the medium as a result of the enzymatic reaction has been presented in Figure 2 for both dyes.

### 3.2. Effect of the Initial Enzyme Concentration

**Figure 3:**
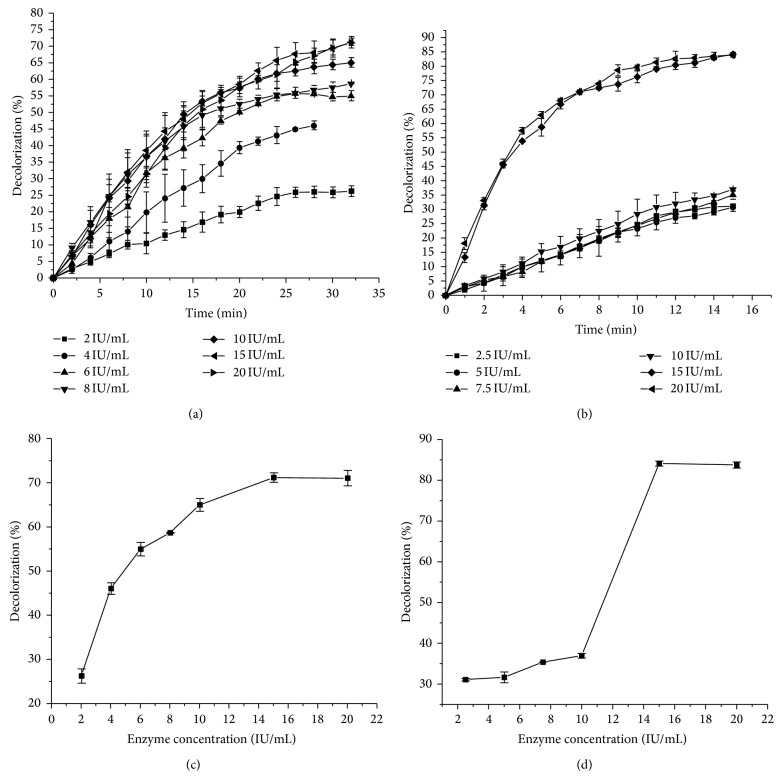
(a) Time course of decolorization of AB 225 and (b) AV 109 dyes at different initial enzyme concentration. (c) The influence of the initial enzyme concentration on the decolorization percentage for AB 225 and (d) AV 109. Reaction conditions for AB 225: treatment time 32 minutes, dye concentration 30 mg/L, hydrogen peroxide concentration 1 mM, pH 5.25, temperature 24°C, and for AV 109: treatment time 15 minutes, dye concentration 30 mg/L, hydrogen peroxide concentration 2 mM, temperature 24°C, pH 5.5. Each value represents the average of three experiments.

Effect of the initial enzyme concentration on the dye decolorization was investigated in the range from 0.02 to 0.2 IU/mL for both dyes and results are presented in Figure 3.

It was apparent that the removal of both dyes increased with an increase in the initial enzyme concentrations. When the concentration reached 0.15 IU/mL, 83.68 and 70.37% of AV 109 and AB 225 were removed, respectively. For the removal of the azo dye, Acid Black 10 BX, the enzyme concentration of 2.205 IU/mL was required which is fifteen times higher than in the case of the anthraquinonic dyes examined in this work [23]. On the other hand, Celebi et al. found out that the necessary enzyme concentration for removal of the anthraquinonic dye, Reactive Blue 19, was 3.30 *μ*g/mL which is almost six times higher than the amount required for AB 225 or AV 109 dyes [7]. In the current research, the HRP concentration of 0.15 IU/mL was sufficient to achieve 83.7% or higher removal of AV 109 and AB 225, within only 32 and 15 minutes of treatment, respectively, suggesting its rather high potential for decolorization of these anthraquinonic dyes.

### 3.3. Initial Hydrogen Peroxide and Dye Concentration Influence

**Figure 4:**
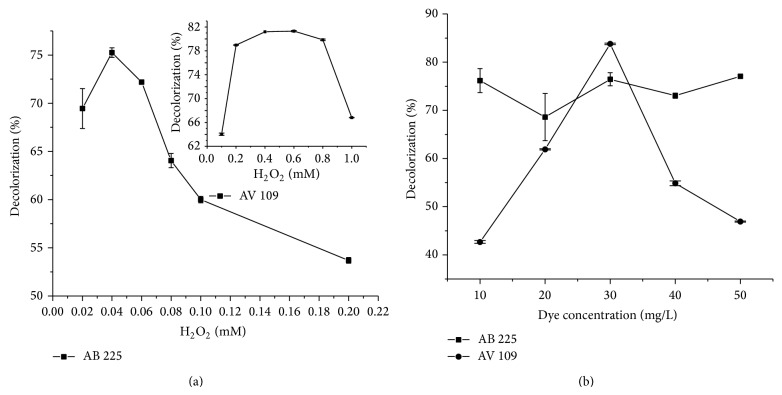
The decolorization percentage as a function of H_2_O_2_ concentration (a) and dye concentration (b). Reaction conditions for AB 225: reaction time 32 minutes, enzyme concentration 0.15 IU/mL, pH 5.25, and temperature 24°C; for AV 109: reaction time 15 minutes, enzyme concentration 0.15 IU/mL, pH 5.5, and temperature 24°C.

To examine the influence of hydrogen peroxide, its concentration was varied in the range from 0.1 to 1.0 and from 0.02 to 0.2 mM for AV 109 and AB 225, respectively. The ranges were selected in the preliminary study which revealed that the oxidation of AV 109 consistently required less hydrogen peroxide in comparison with AB 225. Results are shown in Figure 4(a).

The inhibitory effect of hydrogen peroxide was evident, particularly in the case of AB 225 (Figure 4(a)). Namely, an increase of the dye decolorization percentage was apparent in the hydrogen peroxide concentration range of 0.02–0.04 mM. Further addition of hydrogen peroxide resulted in a decrease of dye removal. Although the requirement of H_2_O_2_ was significantly different from that for AB 225, a similar trend was observed for AV 109 (insert in Figure 4(a)). Namely, an increase of dye removal can be noticed in the hydrogen peroxide concentration range of 0.2–0.6 mM and at higher concentration, the decolorization range decreased. The inhibitory effect of hydrogen peroxide on HRP was also noticed by other researchers. For the removal of C.I. Acid Orange 7 from aqueous solution it was necessary to add hydrogen peroxide up to concentration of 0.8 mM [32]. Similar behavior was noticed when the decolorization of an azo dye, C.I. Acid Blue 25, was studied showing that the optimal concentration of hydrogen peroxide was 0.8 mM. Hydrogen peroxide concentration higher than 0.8 mM resulted in lower percent of dye removal in both cases. In order to avoid the inhibitory effect of hydrogen peroxide, different approaches have been used including the addition of glucose oxidase. Glucose oxidase produces hydrogen peroxide only in dose necessary for the reaction, so inhibitory effect of higher hydrogen peroxide concentration can be avoided [33].

Influence of the substrate concentration (dye) was examined by varying the concentration of dye in the range 10–50 mg/L, and the results are shown in Figure 4(b). It seemed clear that there was also an optimum dye concentration for removal of both anthraquinonic dyes catalyzed by HRP. The optimal reaction values are presented in Table 3. Although there is no apparent substrate inhibition for AB 225, AV 109 seemed to act as a strong inhibitor at investigated conditions. However, at typical environmental dye concentrations, the HRP-catalyzed treatment process seemed also to be effective for this dye.

### 3.4. Initial pH Influence

**Figure 5:**
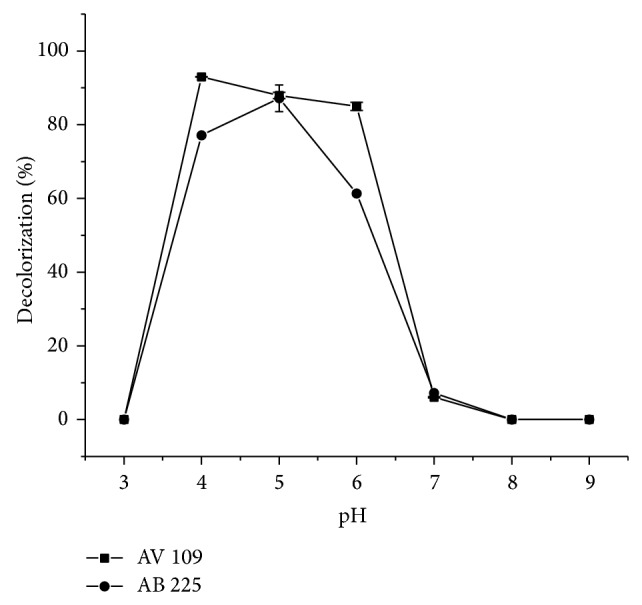
The influence of the initial pH on AB 225 and AV 109 decolorization at 24°C with 0.15 IU/mL HRP. Reaction conditions for AB 225: 32 minutes, dye concentration 30 mg/L, and hydrogen peroxide concentration 0.04 mM; for AV 109: 15 minutes, dye concentration 30 mg/L, and hydrogen peroxide concentration 0.4 mM. Relative standard deviations (RSD) of triplicate measurements were always less than 5%.

To examine influence of pH on decolorization reaction, 0.1 M citrate buffer (pH 3–6) and 0.1 M phosphate buffer (pH 6–9) were used for both dyes (Figure 5).

Figure 5 clearly shows a rather high impact of pH on the enzyme activity. The bell-shaped curves with a defined pH optimum in the range from 4 to 5 were apparent for both dyes. In this pH range, an extremely high dye decolorization of 89.7 and 92.98% has been noticed for AV 109 and AB 225, respectively. This is an advantage from industrial application point of view since some dye effluents are slightly acidic [19]. On the other hand, in the pH range lower than 3.6 or higher than 6, a significant decrease in dye decolorization was observed, which can be attributed to the pH-dependence of HRP activity [34]. Many substrates may have ionizable groups and only one of the ionized forms of the substrate may be acted upon by the enzyme, so one of the reasons why at a pH below 3.6 and above 6 the small amount of dye is decolorized is that substrate is in that form so the enzyme cannot demonstrate its catalytic activity. Another reason can be that on either the basic or the acidic side of this pH optimum, the substantial structure changes in the enzyme may occur, causing a decline in the specific activity of the enzyme. It is well-known that the release of the heme group from the active site of the enzyme is pH dependent, occurring more rapidly below pH 4 and leading to a loss of HRP activity [35]. The results are in contrast to the observations made for HRP-catalyzed degradation of Remazol Blue where an acid pH of 2.0 was found to be optimal, revealing that this azo dye acted as a strong competitive inhibitor of HRP at pH values above 6.0 [36]. Similarly, the decolorization efficiency of methyl orange decreased with the increase of pH value of the reaction mixture above 3.0, due to substrate inhibition at higher pH values [36]. Further experiments in this work were conducted in aqueous solutions with pH 4 and 5 for AV 109 and AB 225, respectively.

### 3.5. Initial Temperature Influence

**Figure 6:**
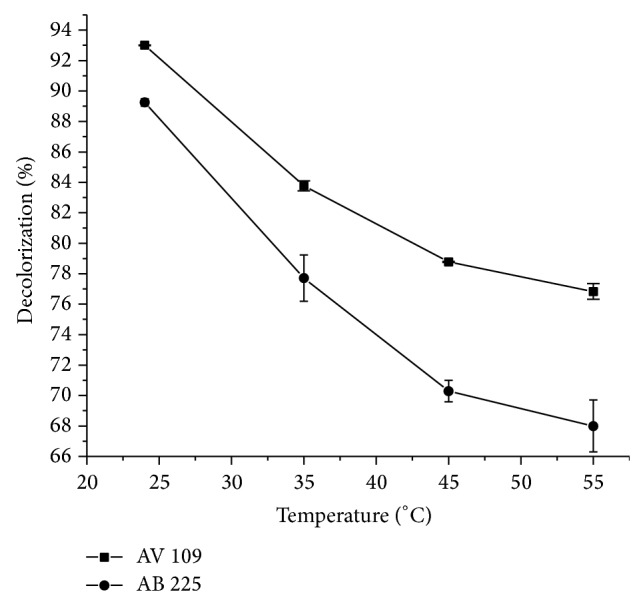
The influence of temperature on AB 225 and AV 109 decolorization. Other reaction conditions were optimal for both dyes.

The influence of temperature on dyes removal was examined at the optimal pH for particular dye in the range 24–55°C (Figure 6). It appeared that the temperature profiles were similar for HRP degradation of both dyes. A typical bell-shaped curve with a defined temperature optimum was not obtained within the range tested. Namely, a rapid decrease in the activity was found with increasing temperature. The optimal temperature for the dye decolorization was accepted to be 24°C. At the end, when the reactions were carried out under optimal conditions (Table 3), 94.7% of the AV 109 and 89.36% of the AB 225 dye were decolorized. The reported values of the optimal temperature for HRP varied, depending on the individual substrate.

### 3.6. The Initial Kinetic Study

HRP-catalyzed oxidation of organic substrates, using H_2_O_2_ (or other peroxides) as electron acceptor could be often described with a bisubstrate ping-pong kinetic model. The model is characterized by the product of the enzyme's reaction with the first substrate (in the case of peroxidases this is the reagent H_2_O_2_) being released before the reaction of the enzyme with the second substrate (anthraquinonic dye, DH). Specifically, H_2_O_2_ binds to the enzyme (E) in the first step of the ping-pong mechanism resulting in the formation of the oxidized form of the enzyme (EI, compound I) and a molecule of water. The produced compound I is then reduced by a hydrogen donor (dye, DH, the second substrate in a ping-pong bi-bi mechanism)* via* a one-electron transfer process to form a second enzyme intermediate called compound II (EII) and a donor radical. Next step in the catalytic cycle of HRP is reduction of compound II again* via* a one-electron transfer process, returning the enzyme to its initial form, ready for the next catalytic cycle, and producing another donor radical [37–39].

The mechanism can be summarized by the following set of equations [25]:

(2) $$\begin{array}{c} \text{E}+{\text{H}}_{2}{\text{O}}_{2}\overset{k_{1}}{\to }\text{EI}+{\text{H}}_{2}\text{O} \end{array}$$

(3) $$\begin{array}{c} \text{EI}+\text{DH}\overset{k_{2}}{\to }\text{EII}+{\text{D}}^{•} \end{array}$$

(4) $$\begin{array}{c} \text{EII}+\text{DH}\overset{k_{3}}{\to }{\text{D}}^{•}+\text{E}+{\text{H}}_{2}\text{O} \end{array}$$

where *k*_1_, *k*_2_ and *k*_3_ are reaction rate constants.

The net HRP-catalyzed reaction is summarized as follows:

(5) $$\begin{array}{c} {\text{H}}_{2}{\text{O}}_{2}+2\text{DH}\overset{\text{HRP}}{\to }2{\text{D}}^{•}+{\text{H}}_{2}\text{O} \end{array}$$

This is the basic ping-pong model and it is well described by means of (6) (kinetic models for ping-pong bi-bi mechanisam with supstrate inhibition).

Ping-Pong mechanisam without inhibition:

(6) $$\begin{array}{c} v_{0}=\frac{V_{\max}{\left[{\text{H}}_{2}{\text{O}}_{2}\right]}_{0}{\left[\text{D}\right]}_{0}}{K_{mb}{\left[{\text{H}}_{2}{\text{O}}_{2}\right]}_{0}+K_{ma}{\left[\text{D}\right]}_{0}+{\left[{\text{H}}_{2}{\text{O}}_{2}\right]}_{0}{\left[\text{D}\right]}_{0}}. \end{array}$$

Ping-Pong mechanism with H_2_O_2_ inhibition:

(7) v0=Vmax⁡H2O20D0 ×KmbH2O201+H2O20Kia   +KmbD0+H2O20D01+H2O20Kia−1.

Ping-Pong mechanism with dye [D] inhibition

(8) v0=Vmax⁡H2O20D0KmbH2O20+KmaD01+D0/Kib+H2O20D0,

*v*_0_—initial rate of the reaction, *V*_max⁡_—maximum rate, [H_2_O_2_]_0_, [D]_0_—initial concentrations of hydrogen peroxide and dye, respectively, *K*_*mb*_, *K*_*ma*_—Michaelis constants for dye and hydrogen peroxide, respectively, *K*_*ia*_, and *K*_*ib*_—inhibition constants for hydrogen peroxide and dye, respectively.

In order to test the validity of the bisubstrate kinetic ping-pong mechanism for the investigated HRP enzyme catalyzed oxidation reaction, the initial kinetic study was performed at previously determined optimum reaction parameters for both dyes. The experimental data obtained in both systems were fitted to a general two-substrate ping-pong kinetic model (three parameters, equation (6)) and a ping-pong kinetic model that includes peroxide inhibition (four parameters, equation (7)) or dye inhibition (four parameters, equation (8)).

**Figure 7:**
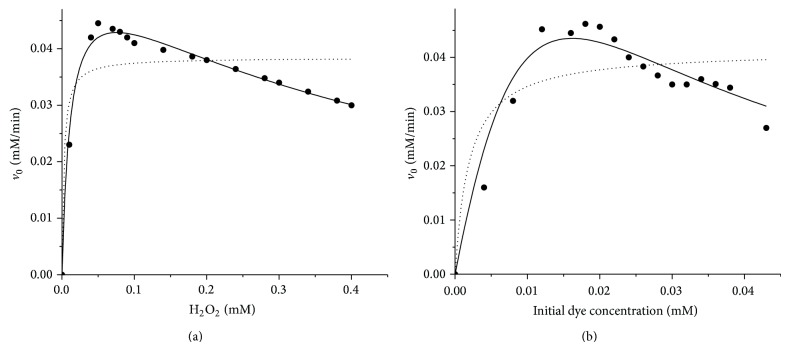
(a) Initial rate* versus* hydrogen peroxide concentration at fixed AV 109 concentration of 0.018 mM and (b) initial rate* versus *AV 109 concentration at fixed hydrogen peroxide concentration of 0.06 mM. Symbols represent experimental data, lines indicate kinetic models based on fitted kinetic parameters including substrate inhibition (solid lines) and without substrate inhibition (dotted lines).

**Figure 8:**
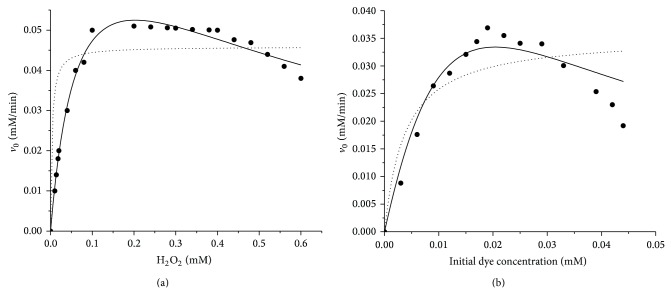
(a) Initial rate* versus *hydrogen peroxide concentration at fixed AB 225 concentration of 0.029 mM and (b) initial rate* versus *AB 225 concentration at fixed hydrogen peroxide concentration of 0.1 mM. Symbols represent experimental data, lines indicate kinetic models based on fitted kinetic parameters including substrate inhibition (solid lines) and without substrate inhibition (dotted lines).

Figures 7 and 8 show the experimentally obtained profiles for AV 109 and AB 225, respectively, and the fitted model values of the initial rate* versus* varying concentrations of one of the substrates, while the other one was kept constant.

The results indicated the presence of inhibition by both substrates. Namely, the experimentally obtained profiles of *V*_max⁡_ against the concentration of both substrates had no a hyperbolic dependence characteristic for the ping-pong kinetic model. By contrast, the initial kinetic data for both dyes showed inhibition with increasing H_2_O_2_ and dye concentration (Figures 7 and 8).

**Scheme 1:**
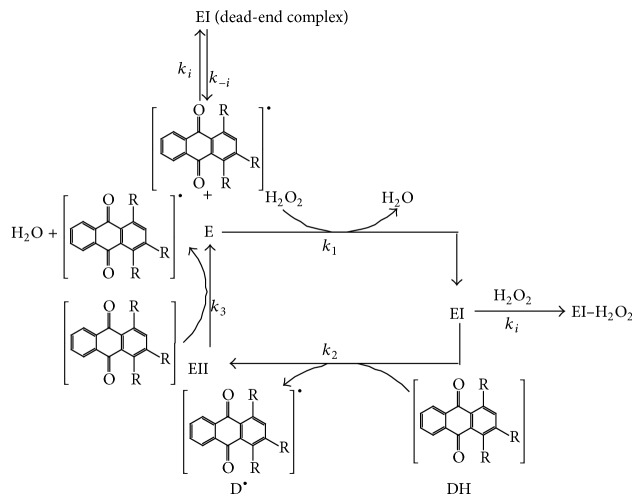
Schematic representation of the ping-pong bi-bi mechanism with dye and H_2_O_2_ inhibition.

Thus, the ping-pong kinetic model that includes inhibition, by forming a dead-end complex, has been proposed. According to this mechanism, since the three forms of the enzyme—E, compound I and compound II have a similar structure, it is reasonable to expect that dye DH has some affinity for E as well as for compounds I and II and or H_2_O_2_ also can show some affinity for compounds I or II, as shown on Scheme 1.

The best fit for experimental data obtained for both dyes was achieved when the ping-pong bi-bi mechanism with H_2_O_2_ inhibition was used, revealing the permanent enzyme inactivation in the presence of excess H_2_O_2_. The model concurred very well with the experimental data (*R* ^2^ = 0.9902 and 0.9908 for AV 109 and AB 225 resp.). However, the basic ping-pong model seemed to underpredict the rate of dyes removal as rather high deviations of the experimental kinetic data from the theoretical fitted value were obtained for both dyes.

The slightly lower *K*_*m*_ value for AB 225 suggested that HRP had higher apparent affinity toward this dye. However, from the inhibition constants values (*K*_*i*_) shown in Table 4, it can be concluded that the AV 109 dye exhibited a rather high inhibitory effect on HRP enzyme. By analyzing the values of the inhibition constant for dye (0.008 mM) and inhibition constant for hydrogen peroxide (0.4436 mM), it can be concluded that in the case of AV 109, dye has stronger inhibitory effect on HRP enzyme. On the other hand, AB 225 showed similar effect. Namely, inhibition constant is 0.01259 mM for dye, and 0.4767 mM for hydrogen peroxide. However, by comparing the values of the inhibition constants of each of the examined dyes, it can be concluded that AV 109 is significantly higher potent inhibitor than AB 225. These data were consistent with the previous optimization study, where a higher AV 109 concentration caused a sharp decline in the day decolorization. Ping-pong reaction kinetics has also been observed for several other peroxidase-catalyzed oxidations mediated by compound I; however, the inhibition by both substrates was often neglected.

### 3.7. COD and TOC

COD and TOC can be used to indirectly determine the amount of organic matter in water and wastewater and thus can be applied for evaluation of the efficiency of water treatment. The initial value of COD for AB 225 sample was 633 mg O_2_/L. After the decolorization experiments, COD was determined to be 493 mg O_2_/L (decrease by 22%). Similar behavior was noticed in the case of AV 109. Namely, the sample had an initial value of 668 mg O_2_/L, and after the treatment there was a decrease in COD value to 488 O_2_/L (decrease by 26%). All these results showed that decolorization had influence on COD reduction. A strong correlation between the values of chemical oxygen demand and total organic carbon appeared. Namely, TOC analysis showed similar behavior and the value of total organic oxygen decreased by 14 and 38% for AV 109 and AB 225, respectively.

## 4. Conclusion

It appears that HRP is a promising biocatalyst for the oxidative decolorization of anthraquinonic dyes. It was capable of achieving 94.7% removal of AV 109 and 89.36% removal of AB 225 under optimal conditions after 15 and 32 min of treatment, respectively. The kinetic analysis was carried out at optimum operating conditions, revealing that the experimental kinetic data was adequately modelled by the ping-pong bi-bi kinetics with substrate inhibition. Overall, the high affinity of the enzyme to both dyes and a rather high percentage of decolorization, the pH optimum occurring in the range of some slightly acidic dye effluents have important implications for the potential of HRP in degradation of anthraquinonic dyes. However, the inactivation of the enzyme in the presence of the dyes can be the major limitation in potential commercial application of the technique for anthraquinonic dye removal. Thus, additional investigations and development could be carried out to improve the decolorization process, including immobilization of the enzyme and its application for real industrial substrate decolorization in the presence of competitive matters.

## Figures and Tables

**Table 1:** Analyzed anthraquinonic dyes properties.

Name: C.I. Acid Violet 109 Molecular structure: anthraquinones Molecular formula: C_35_H_34_Br_2_NaO_7_S Molecular weight: 823.52 g/mol CAS registry number: 12220-63-2 *λ*_max⁡_: 590; 552 nm	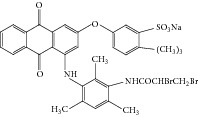

Name: C.I. Acid Blue 225 Molecular structure: anthraquinones Molecular formula: C_26_H_20_Br_2_N_3_O_6_S Molecular weight: 685.32 g/mol CAS registry number: 12216-97-6 *λ*_max⁡_: 628; 589 nm	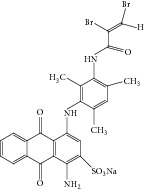

**Table 2:** The investigated range of the reaction parameters selected for the optimization of dyes decolorization.

Parameters	AV 109	AB 225
pH	3.0–9.0	3.0–9.0
Temperature, °C	24–55	24–55
Hydrogen peroxide concentration, mM	0.1–1.0	0.02–0.2
Dye concentration, mg/L	10–50	10–50
Enzyme concentration, IU/mL	0.02–0.2	0.02–0.2

**Table 3:** Optimal parameters for examined anthraquinonic dyes decolorization by HRP.

Optimal	AV 109	AB 225
pH	4.0	5.0
Temperature, °C	24	24
Hydrogen peroxide concentration, mM	0.4	0.04
Dye concentration, mg/L	30	30
Enzyme concentration, IU/mL	0.15	0.15

**Table 4:** Best fit of kinetic parameters obtained for the H_2_O_2_-mediated oxidation of AV 109 and AB 225 by HRP modeled by ping-pong bi-bi model considering inhibition with substrates and without inhibition.

Kinetic parameters	Dye inhibition	Without dye inhibition	Hydrogen peroxide inhibition	Without hydrogen peroxide inhibition
AV 109				
*V*_max⁡_ (mMmin^−1^)	1.638	0.5178	1.097	1.185
*K*_*m*D_ (mM)	0.2374	0.0245	0.3302	0.5373
*K*_*i*_ (mM)	0.0080	/	0.4436	/
*K*_*m*H_2_O_2__ (mM)	0.4372	0.6908	0.2356	0.8081
*R* ^2^	0.9225	0.6456	0.9902	0.7520
AB 225				
*V*_max⁡_ (mMmin^−1^)	0.9813	0.3266	0.5625	1.006
*K*_*m*D_ (mM)	0.2213	0.03464	0.1522	0.6069
*K*_*i*_ (mM)	0.0126	/	0.4767	/
*K*_*m*H_2_O_2__ (mM)	0.6685	0.8203	0.4536	0.0723
*R* ^2^	0.9602	0.7730	0.9908	0.9067

*v*_0_—initial rate of the reaction, *V*_max⁡_—maximum rate, [H_2_O_2_]_0_ and [D]_0_—initial concentrations of hydrogen peroxide and dye, respectively, *K*_*mb*_, *K*_*ma*_—Michaelis constants for dye and hydrogen peroxide, respectively, *K*_*ia*_, *K*_*ib*_—inhibition constants for hydrogen peroxide and dye, respectively.
